# 733 Massive Burn Injuries: Characteristics, Treatment Strategies and Outcomes from a Single Institution

**DOI:** 10.1093/jbcr/irac012.287

**Published:** 2022-03-23

**Authors:** Jason Heard, David L Wallace, Kathleen S Skipton Romanowski, Tina L Palmieri, David G Greenhalgh, Soman Sen

**Affiliations:** University of California - Davis, Sacramento, California; University of California, Davis, Sacramento, California; UC Davis, Sacramento, California; UC Davis and Shriners Hospitals for Children Northern California, Sacramento, California; University of California - Davis, Sacramento, California; UC Davis, Sacramento, California

## Abstract

**Introduction:**

Advances in burn care have led to improved survival, with survival after a 50% total body surface area (TBSA) or larger burn being more common. Traditionally, age, TBSA burned, inhalation injury, delayed resuscitation and evidence of early organ dysfunction have been predictive of survival. The goal of this study was to describe a large series of massive burn injuries, treatment strategies and identify factors related to survival.

**Methods:**

Following IRB approval, a retrospective review of adult patients who sustained 50% TBSA or larger burn from 8/2009 to 7/2019 at an ABA verified burn center was conducted. Demographic, burn size/depth, mechanisms of injury, treatments, and outcome data were collected. Univariate and multivariate analyses were performed using R statistical software (R-project.org).

**Results:**

155 patients were included which was 4.7% (155/3312) of all burn admissions during that time internal. Patients had an average age of 44±18 years, a male predominance (79%), and average TBSA burned of 70±15%. Overall mortality was 54% (83/155). One third of patients were transitioned to comfort care. The 103 treated patients were younger (37±12 vs 59±19 years; p=< 0.0001), more likely to be male (85 vs 65%; p=0.006), had smaller average TBSA (66±13 vs 78±16%; p< 0.0001) and more likely to have a psychiatric condition (31 vs 13%; p=0.02). Approximately 70% of treated patients survived to discharge. Survivors were more likely to have smaller TBSA (63±13 vs 73±13; p=0.001) and less third-degree burns (49±24 vs 61±24; p=0.01). One third of treated patients developed renal failure. One quarter of patients had a mental health condition, and these patients spent more time in the hospital (61 vs 31 days; p=0.009), more time on ventilator (29 vs 12 days; p=0.046), required more surgery (3 vs 2; p=0.048), and were less likely to die (36% vs 59%; p=0.02). On multivariate regression analysis of treated patients, psychiatric illness (OR 0.19; p=0.03) and burns related marijuana/hash oil production (OR 0.13; p=0.015) were protective against mortality.

**Conclusions:**

Surviving burns >50% TBSA is becoming more common as burn care continues to improve. Mortality in this study is lower than what would be predicted by an established revised Baux score regression model (predicted 61% overall mortality and 48% for treated). Care for these massive burn injuries is complex and requires an experienced multidisciplinary team. There is an established link between burn injuries and mental health conditions. Despite similar burn size/depth, patients with a mental health history spent significantly more time in the hospital, more time on the ventilator and required more surgery.

